# Supplementary figures and images for: CASPULE: A computational tool to study sticker spacer polymer condensates
Source: PLoS Comput Biol. 2026 May 14;22(5):e1014282. doi: 10.1371/journal.pcbi.1014282 (PMC13186343; doi:10.1371/journal.pcbi.1014282)

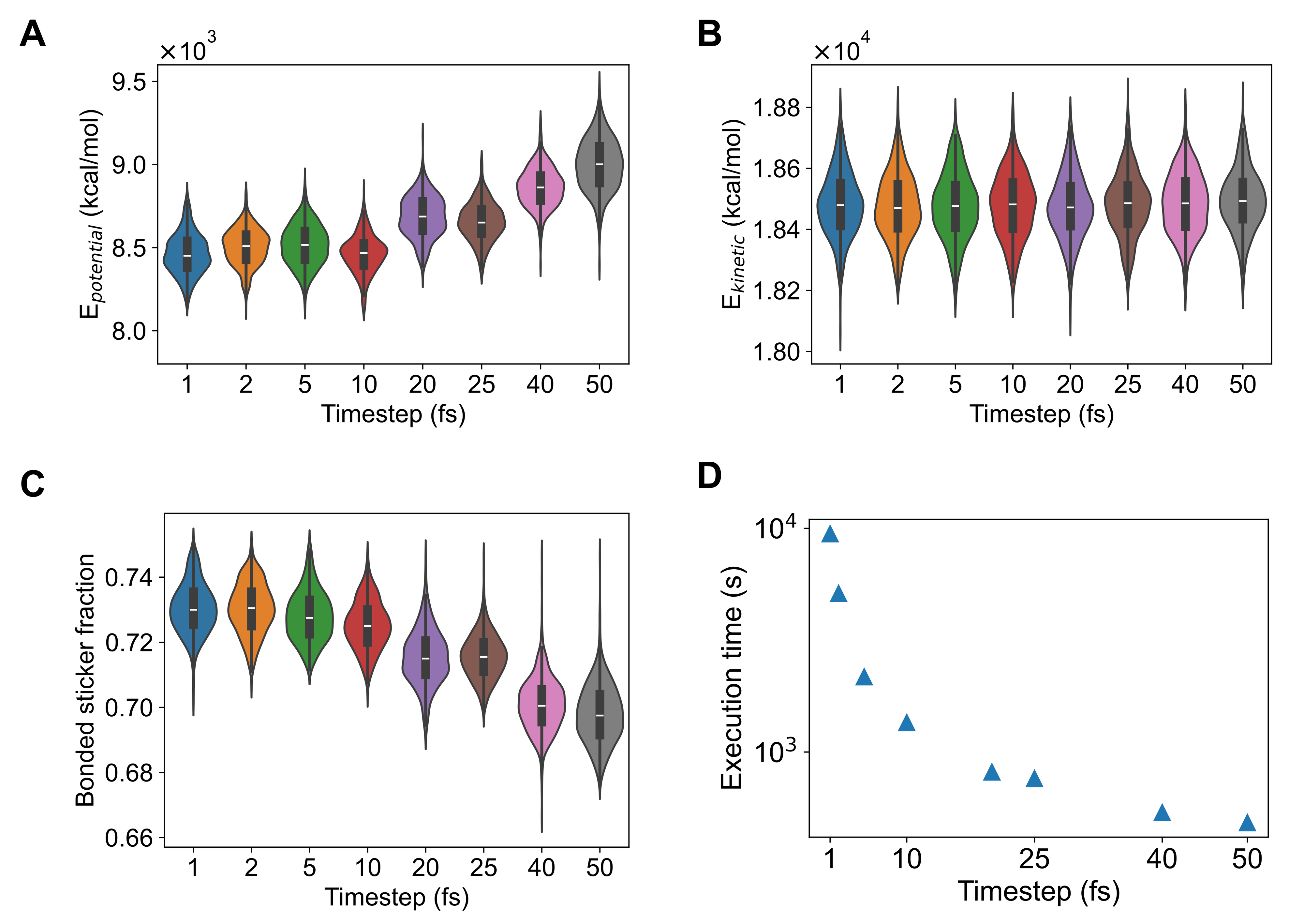

Supplement: S1 Fig — Trend of (A) potential energy (B) Kinetic energy (C) Sticker bonding statistics and (D) execution time. Energy parameter, Es = 6kT, Ens = 0.3kT. Damping parameter = 200 fs. Total elapsed or simulation time = 50 * 106. (TIF) [file pcbi.1014282.s001.TIF]

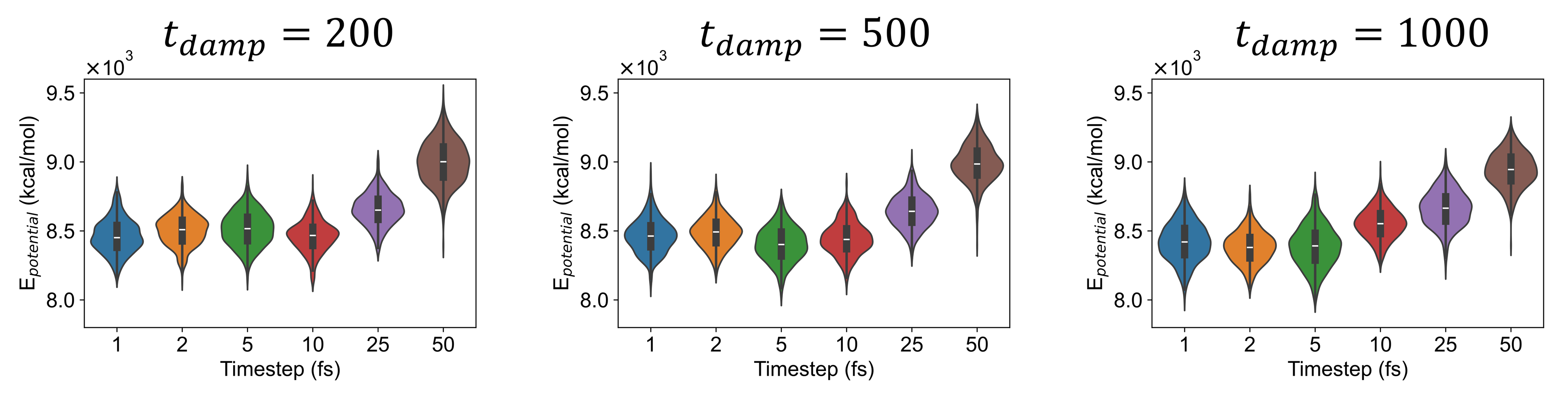

Supplement: S2 Fig — Trend of the potential energy at three different values of damping time. Energy parameter, Es = 6kT, Ens = 0.3kT. Damping parameter = 200 fs. Total elapsed or simulation time = 50 * 106. (TIF) [file pcbi.1014282.s002.TIF]

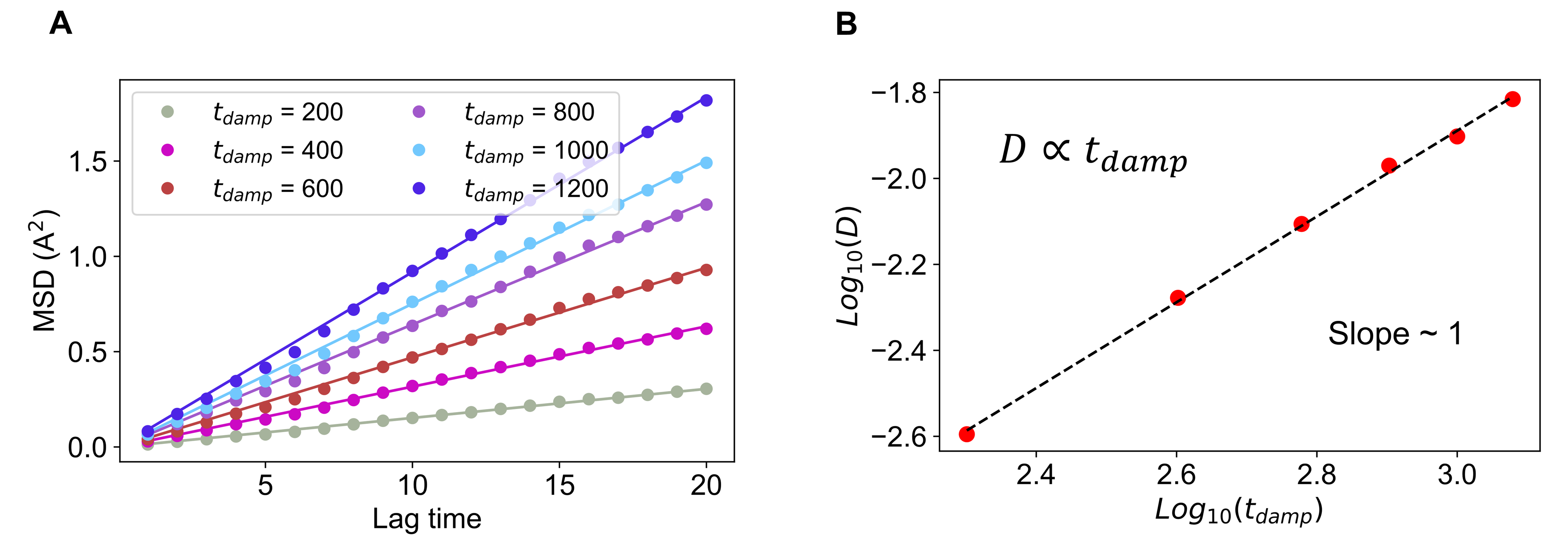

Supplement: S3 Fig — (A) Mean squared displacement vs. elapsed time plot for six different damping time (viscosity). The circles indicate actual displacements, and solid lines show the linear fit. Diffusion coefficient, D, is extracted from the linear fit: MSD = 6Dt (B) D vs. damping time in log-log scale. The red points are extracted Ds from MSD trajectories. The black dashed line is the linear fit which yields a slope ~ 1. In these simulations, timestep = 10 fs. Energy parameters, Es = 6kT, Ens = 0.3kT. (TIF) [file pcbi.1014282.s003.TIF]
