# Supplementary material for: CASPULE: A computational tool to study sticker spacer polymer condensates
Source: PLoS Comput Biol. 2026 May 14;22(5):e1014282. doi: 10.1371/journal.pcbi.1014282 (PMC13186343; doi:10.1371/journal.pcbi.1014282)
Supplement: Attachment — Submitted filename: Response_Letter_CASPULE.pdf [file pcbi.1014282.s006.pdf]

## Rebuttal Letter

CASPULE: A computational tool to study sticker spacer polymer condensates

PCOMPBIOL-D-25-02356

**Black: Reviewers' comments, Blue: Our response**

We thank all the reviewers for their careful reading and constructive feedback. To address the collective concerns, in the revised manuscript,

- we have added one new figure (Figure 3) that demonstrates the validity of detailed balance in "one-to-one" sticker bonding scheme
- we provided 3 supplementary figures (Figs. S1,S2,S3) showing the effect of timestep and damping factors in the accuracy and computational cost of our simulations
- we also majorly updated the documentation related to installation of CASPULE that automates the setup process.

The modified sections are highlighted in yellow in the revised manuscript. We hope that these additional results will satisfy the reviewers' concerns adequately. A pointwise rebuttal is given below.

**Reviewer 1:**

The authors describe a simulation pipeline to perform off-lattice sticker-spacer simulations of binary heteropolymer mixtures, in which reactive beads on one polymer species can form "specific" one-to-one bonds with reactive beads on the other polymer species. Although simulations of this system by this group and others (e.g., Pappu, Wingreen) have been reported previously, including using this off-lattice approach, the present manuscript describes the steps required to set up and run such simulations clearly. It therefore reads as a user-friendly extended methods section for previous papers on this system from this group. Overall, the manuscript is well organized and easy to read.

We thank the reviewer for their positive assessment.

I have two scientific concerns regarding the methodology:

1. It is not clear that the "specific interaction" scheme, which is based on "fix bond/break", satisfies detailed balance. First, the authors should provide a proof, both because this property is essential for any simulation method and also because the pedagogical nature of this methods-oriented manuscript requires that all algorithmic details are completely explained. Second, the authors should show that the results (e.g., phase boundaries) are insensitive to the time step and damping factor in the Langevin simulation. Third, the authors should show that these results are invariant to changes in the integration scheme, e.g., to Nose-Hoover or even to a Metropolis Monte Carlo algorithm. Finally, the authors should show that no issues arise from combining Monte Carlo bond breaking/formation moves with Langevin dynamics, since it is well known that subtle problems arise from non-area-preserving integration schemes (see, e.g.,

Bussi, Giovanni, and Michele Parrinello. "Accurate sampling using Langevin dynamics." Physical Review E—Statistical, Nonlinear, and Soft Matter Physics 75.5 (2007): 056707.).

The newly added figure (Figure 3) and related result section shows proof of the detailed balance. The newly added supplementary information now contains a discussion about the effect of timestep and damping on simulation results. Fig. S1 shows the impact of timestep on simulation results. In a nutshell, a timestep of 10-30 fs (real unit, LAMMPS) provides a good tradeoff between numerical accuracy and computational cost. Fig. S2 shows that higher viscosity (smaller $t_{damp}$ ) allows for a larger timestep tolerance and vice versa. Importantly, we added Fig. S3 to show the expected relationship between diffusion coefficient and damping factor (viscosity) that are important to capture the kinetics of the system.

Validating Langevin integrator with other types of integration scheme feels out of scope for the current manuscript. Since LAMMPS is a mature and advanced software widely used by many scientific communities, we can rely on the numerical accuracy of the solver.

Regarding combining MC and LD, we now provide a detailed balance test for two-particle system. We also provide benchmark simulations related to the accuracy of sampling the energetics (equilibrium) as a function of timestep and viscosity. Users may choose the respective parameters for future references.

2. It is standard practice to use slab-geometry direct-coexistence simulations to compute phase boundaries, since the curved interfaces of finite-size droplets can modify the coexistence conditions due to a non-zero Laplace pressure. Since the manuscript discusses the calculation of phase boundaries, results using the slab geometry should be presented.

In the revised version, we highlighted that, through CASPULE, we want to capture the kinetic effects that may govern condensate size distribution and internal structures. To that end, slab geometry is not ideal as the diffusion timescale gets artificially manipulated in this modified aspect ratio of the simulation box. It is a convenient strategy to capture the equilibrium behavior (phase co-existence, for instance) of the system. However, kinetic factors needed to capture condensate ageing and size control cannot be properly accounted for in a slab-like geometry. Hence, we prefer to use standard cubic geometry in our sticker spacer simulations.

**Reviewer 2:**

This manuscript presents CASPULE, a computational pipeline for simulating sticker–spacer polymers with strict single-valent bonding using Langevin dynamics in LAMMPS. Overall, I find this to be a reasonable and potentially useful contribution. The work clearly demonstrates how limited-valency interactions can be incorporated into particle-based simulations, and such interactions are increasingly recognized as important for modeling biomolecular condensates. Compared to existing lattice-based or Monte Carlo approaches, the ability to access dynamical information is a notable strength and could be valuable for studying kinetic arrest, coarsening, and aging phenomena in condensates.

We thank the reviewer for their positive assessment.

That said, in its current form the project feels closer to a detailed simulation protocol than to a polished, user-friendly software package. While the documentation is thorough, the setup requires users to navigate multiple external tools (e.g., Moltemplate, PACKMOL, custom LAMMPS scripts) and manually coordinate several steps. This creates a fairly high barrier to entry. Streamlining the workflow, ideally by wrapping the setup and execution steps into a unified Python interface, would substantially increase the accessibility and impact of CASPULE for the broader community.

Thanks for this suggestion. We significantly modified the installation section of the documentation. Now we provide an installation script that streamlines most of the third-party package (Moltemplate, packmol) installations (including LAMMPS download and build, adding bond/create/random automatically) and creates a virtual environment that is ready to run CASPULE simulations. We also include necessary python packages to run the data analysis scripts.

In addition, the manuscript emphasizes access to dynamical behavior, but the reversible bonding scheme relies on fix bond/create and fix bond/break in LAMMPS. To my knowledge, these fixes do not rigorously satisfy detailed balance, and therefore do not guarantee that the resulting dynamics (or even steady-state distributions) are thermodynamically consistent. This does not invalidate the approach, but it does raise important questions about how faithfully the reported dynamics correspond to an underlying physical model. A more explicit discussion of this issue, along with its possible implications for interpreting kinetic rates, bond lifetimes, and steady-state properties, would strengthen the manuscript and help readers better understand the scope and limitations of the method.

The newly added figure (Figure 3) and related result section shows proof of the detailed balance. Results from Fig. 4D can also be interpreted as a validation of the detailed balance for multi-chain system which we now have highlighted.

Overall, I believe CASPULE has clear potential, but addressing the points above would significantly improve both its usability and the clarity of its physical interpretation.

**Reviewer 3:**

The author provide a tool that should be useful for simulations of phase separation. Although I have not testet it extensively it seems to work well, documentation is nice and the paper is well written

We thank the reviewer for their positive assessment.
